# Identification of single-nucleotide variants associated with susceptibility to *Salmonella* in pigs using a genome-wide association approach

**DOI:** 10.1186/s12917-020-02344-0

**Published:** 2020-05-15

**Authors:** Corinne H. Schut, Abdolvahab Farzan, Russell S. Fraser, Margaret H. Ainslie-Garcia, Robert M. Friendship, Brandon N. Lillie

**Affiliations:** 1 Department of Pathobiology, University of Guelph, 50 Stone Rd E, Guelph, ON N1G 2W1 Canada; 2 Department of Population Medicine, University of Guelph, Guelph, Ontario Canada; 3 Present address: Department of Pathology and Microbiology, Atlantic Veterinary College, University of PEI, Charlottetown, Prince Edward Island Canada

**Keywords:** *Salmonella*, Swine, Antibody response, Shedding, GWAS, Single-nucleotide variant

## Abstract

**Background:**

*Salmonella enterica* serovars are a major cause of foodborne illness and have a substantial impact on global human health. In Canada, *Salmonella* is commonly found on swine farms and the increasing concern about drug use and antimicrobial resistance associated with *Salmonella* has promoted research into alternative control methods, including selecting for pig genotypes associated with resistance to *Salmonella.* The objective of this study was to identify single-nucleotide variants in the pig genome associated with *Salmonella* susceptibility using a genome-wide association approach. Repeated blood and fecal samples were collected from 809 pigs in 14 groups on farms and tonsils and lymph nodes were collected at slaughter. Sera were analyzed for *Salmonella* IgG antibodies by ELISA and feces and tissues were cultured for *Salmonella*. Pig DNA was genotyped using a custom 54 K single-nucleotide variant oligo array and logistic mixed-models used to identify SNVs associated with IgG seropositivity, shedding, and tissue colonization.

**Results:**

Variants in/near *PTPRJ* (*p* = 0.0000066), *ST6GALNAC3* (*p* = 0.0000099), and *DCDC2C* (*n* = 3, *p* < 0.0000086) were associated with susceptibility to *Salmonella*, while variants near *AKAP12* (*n* = 3, *p* < 0.0000358) and in *RALGAPA2* (*p* = 0.0000760) may be associated with susceptibility.

**Conclusions:**

Further study of the variants and genes identified may improve our understanding of neutrophil recruitment, intracellular killing of bacteria, and/or susceptibility to *Salmonella* and may help future efforts to reduce *Salmonella* on-farm through genetic approaches.

## Background

*Salmonella* is one of the leading causes of foodborne illness and has a significant impact on human health both globally and in Canada [1–3]. While eggs and poultry are the most frequently identified sources of human salmonellosis, pork is also a notable source of *Salmonella* [4–7]. Studies assessing *Salmonella* prevalence through serology and/or culture have frequently identified *Salmonella* in pigs in North America and Europe [8–12]. In pigs, *S.* Choleraesuis infection typically manifests as swine typhoid that may result in diarrhea, fever, and septicemia, similar to human-infecting typhoidal *Salmonella* serovars like *S.* Typhi [13]. Pigs showing visible signs of illness may be treated or removed from the herd to reduce the spread of *Salmonella*. However, the most frequently identified serovars on Canadian swine farms included *S.* Typhimurium, *S.* Typhimurium var. Copenhagen, and *S.* Infantis [9, 14, 15] which typically result in an asymptomatic carrier state in pigs but are known to cause illness in humans [16]. Pigs carrying *Salmonella* asymptomatically play a significant role in on-farm transmission of *Salmonella* within the herd and may limit the effectiveness of control measures implemented on-farm [12]. On-farm control of *Salmonella* has consisted of stringent biosecurity and sanitation practices, as well as the use of antibiotics, vaccination, and quarantine or culling of infected pigs [17–20]. However, the limited effectiveness of these measures in practice has prompted research into swine genetics as a potential alternative measure to control *Salmonella* on swine farms.

Traditionally, selective breeding in swine was established to promote desired production traits including growth performance, feed efficiency, fertility, and meat quality [21–23]. However, with the completion and continued updates to the porcine genome, many studies are now investigating the genetic basis of disease susceptibility in swine. One approach in using genetics to improve resistance is to observe immune traits or phenotypes individually (for example; cytokine production, leukocyte proliferation, and serum levels of IgG or acute phase proteins) [24–27]. Differences in these immune traits and disease severity between pigs and between breeding lines has been well documented which suggests the potential of selective breeding for improved resistance in the near future [19, 24, 28–30]. One such study found that piglets with improved recruitment and function of polymorphonuclear neutrophils, but a lower antibody response, were more resistant to *Salmonella* [28]. As such, it may be possible to select from these breeding lines with more robust immune response phenotypes or desired response traits to promote broad immunity to *Salmonella* in offspring.

Beyond the assessment of immune traits, several studies in recent years have identified significant associations between single-nucleotide variants (SNVs) and/or candidate genes and susceptibility to *Salmonella* in pigs. Candidate gene studies have observed variants in porcine toll-like receptor (TLR) genes that were associated with *Salmonella* fecal shedding [31], and attenuated responses to *Salmonella* Choleraesuis [32]. Upregulation of *TLR5* and *TLR9* has been shown in response to *S.* Choleraesuis and *S.* Typhimurium though its direct impact on *Salmonella* susceptibility is unknown [33]. Additionally, SNVs in mannan-binding lectin (*MBL*) 1 and 2, have been found at higher frequencies in pig populations infected with *S*. Typhimurium and other pathogens [34]. Further, a candidate gene study of the pigs included in this study identified an *MBL1* variant associated with increased *Salmonella* shedding and a variant in *NOD1* associated with isolation of *Salmonella* at slaughter [35].

The candidate gene studies may offer insight into pig susceptibility to *Salmonella* on-farm and at slaughter and benefit efforts in breeding for resistance to common pathogens on-farm. However, a major drawback of candidate gene studies is that they require a priori knowledge of these genes and their functions, and there is still much that is unknown about the pig immune response and the complex interplay between pathogen and host [36]. With recent technological advancements improving the feasibility of genome-wide association studies (GWAS), we can potentially identify novel variants associated with resistance to *Salmonella* shedding and colonization across the entire genome [37]. This study aimed to identify SNVs associated with *Salmonella* IgG antibody response from the end of nursery to market, *Salmonella* shedding from weaning to market, and presence of *Salmonella* in tonsil and lymph node tissues at slaughter in commercial pigs using a GWAS approach.

## Results

Of the pigs included in the GWAS seropositivity model, 32.3% (254/786) of pigs were seropositive at least once from the end of the nursery stage to the end of the finisher stage, for the shedding model 34.2% (269/786) of pigs shed at least once from weaning to end of finisher, and for colonization model 21.6% (111/515) of pigs tested positive for *Salmonella* at slaughter. *Salmonella* positivity for each trait is shown in Table 1.

**Table 1:** Demographics in pigs positive and negative for the trait of interest after quality control of genotypic data

Phenotype	Number of negative pigs (controls*)	Number of positive pigs (cases*)
*Salmonella* seropositivity	254 (32.3%)	532 (67.7%)
*Salmonella* shedding	269 (34.2%)	517 (65.8%)
*Salmonella* isolation at slaughter	111 (21.6%)	404 (78.4%)

*Control: never tested seropositive, never shed *Salmonella*, or tested negative at slaughter; Case: tested seropositive for *Salmonella* at least once, shed *Salmonella* at least once on-farm, or was positive for *Salmonella* at slaughter

### Genome-wide association study

**Fig. 1:**
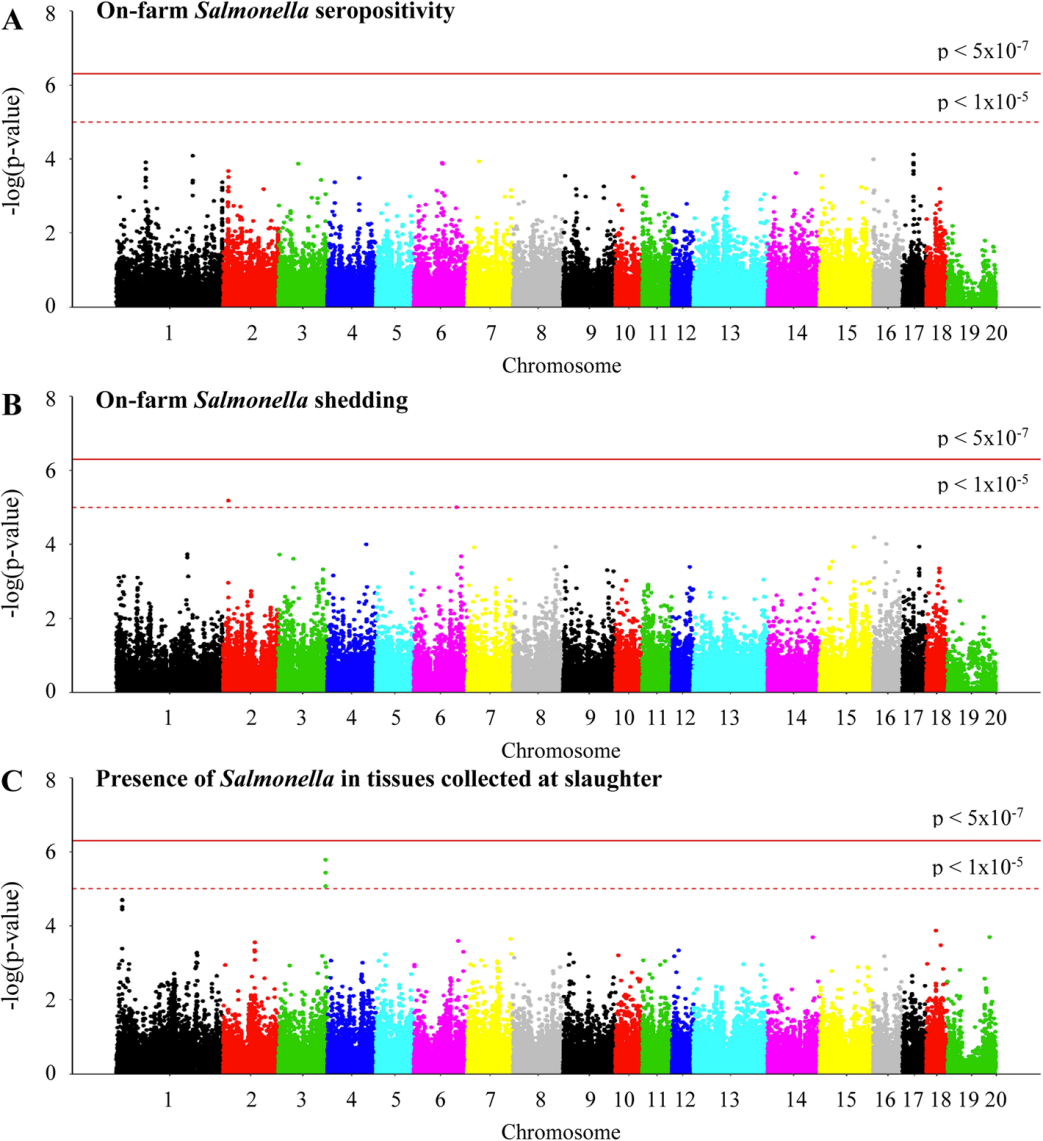
Manhattan plots of the GWAS analysis for *Salmonella* seropositivity from end of nursery to end of finisher (**a**), *Salmonella* shedding from weaning to end of finisher (**b**), and *Salmonella* isolation from tissues at slaughter (**c**). The horizontal solid and dashed red lines indicate the genome-wide threshold for significant (*p* = 5.0 × 10^− 7^) and suggestive (*p* = 1.0 × 10^− 5^) associations, respectively [38]

**Fig. 2:**
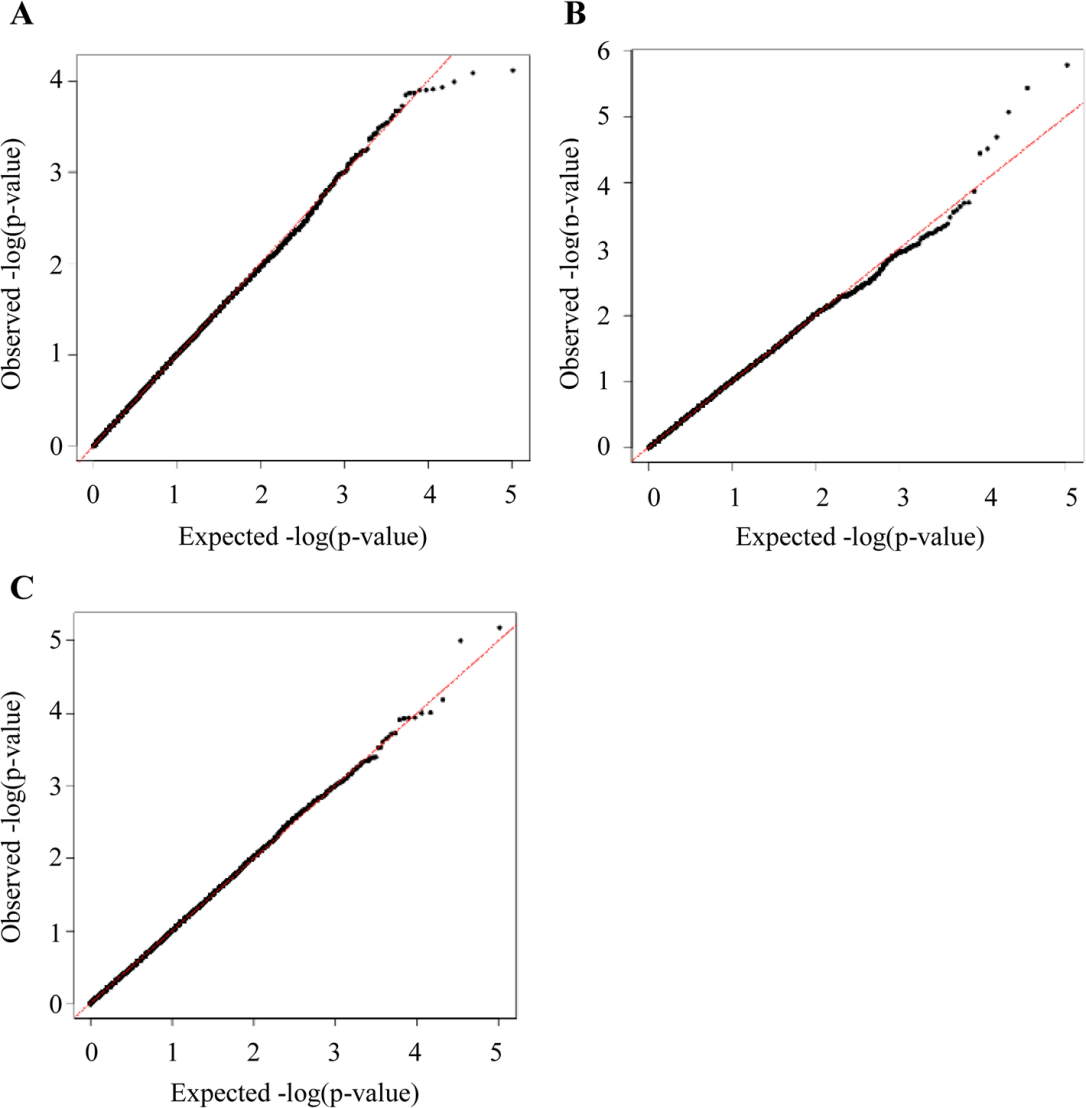
Quantile-quantile plots for *Salmonella* seropositivity from end of nursery to end of finisher (**a**), *Salmonella* shedding from weaning to end of finisher (**b**), and *Salmonella* isolation from tissues at slaughter (**c**). λ = the average genomic inflation factor

After quality control filtering of the data, 51,969 SNVs remained for GWAS analysis from 786 pigs in the seropositivity and shedding models and 515 pigs in the slaughter model. Manhattan plots and quantile-quantile plots for the seropositivity, shedding, and colonization phenotypes are shown in Figs. 1, and 2, respectively. The allele frequencies between case and control pigs for the five significant SNVs and SNVs approaching significance in the GWAS analysis can be seen in the Supplementary Table 1.

Analysis of the shedding trait identified an intron variant in the gene *PTPRJ* on chromosome 2 (*p* = 6.6 × 10^− 6^) as well as an intergenic variant on chromosome 6, downstream of the *ST6GALNAC3* gene (*p* = 9.9 × 10^− 6^), that had a suggestive association (Table 2) and were observed at a higher frequency in the pigs that did not shed *Salmonella*. GWAS analysis of *Salmonella* isolation at slaughter indicated three intergenic SNVs downstream of the gene *DCDC2C* on chromosome 3 (*p* = 1.6 × 10^− 6^, 3.7 × 10^− 6^, 8.6 × 10^− 6^, respectively) under the suggested association threshold of 5.0 × 10^− 5^ (Table 3), two of which were seen at higher frequency in pigs that tested negative at slaughter, while one was more common in pigs positive at slaughter. Three intergenic SNVs on chromosome 1, upstream of the gene *AKAP12* (*p* = 2.0 × 10^− 5^, 3.1 × 10^− 5^, 3.6 × 10^− 5^, respectively), also approached the suggested association threshold and were more common in the pigs that were negative at slaughter. An intron SNV located in *AKAP12* was also observed in the top 15 SNVs for the slaughter model (Table 3). No SNVs were under the suggestive association threshold of 5.0 × 10^− 5^ for the analysis of the seropositivity trait. An intron variant located within the *RALGAPA2* gene on chromosome 17 was the most significant SNV associated with a pig being seropositive at least once from nursery to finisher stage (*p* = 7.6 × 10^− 5^). Additionally, three intron SNVs in the *RALGAPA2* gene were observed within the top 15 significant SNVs for the seropositivity model (Table 4).

**Table 2:** The top 15 SNVs ranked by significance (*p* value) for the GWAS analysis of *Salmonella* shedding from weaning to end of finisher

SNV ID	SSC^**a**^	Location (bp)^**b**^	Variant type	Gene^**c**^	Gene location^**b**^	***p***-value^**d**^
rs324041697	2	14,596,159	intron	*PTPRJ*	2: 14549537–14,726,715	**6.6 × 10**^**−6**^
rs81476180	6	136,607,113	intergenic	*ST6GALNAC3*	6: 136633437–137,201,213	**9.9 × 10**^**−6**^
rs81259485	16	3,248,143	intron	*DNAH5*	16: 3116189–3,364,956	6.6 × 10^− 5^
rs81458700	16	36,370,940	intergenic	*ACTBL2*	16: 36470503–36,471,633	9.9 × 10^− 5^
rs80889714	4	104,920,198	intron	*CASQ2*	4: 104918501–104,982,852	1.0 × 10^− 4^
rs81346930	17	44,032,145	intergenic	*LPIN3*	17: 43993710–44,006,188	1.2 × 10^−4^
rs330624049	15	90,476,754	intergenic	*ENSSSCG00000034554*	15: 90309385–90,309,702	1.2 × 10^−4^
rs81403742	8	117,467,555	intergenic	*TACR3*	8: 117661792–117,721,591	1.2 × 10^−4^
rs80947769	7	19,456,263	upstream	*KIAA0319*	7: 19386111–19,451,666	1.2 × 10^−4^
rs339478050	1	188,917,080	intron	*RTN1*	1: 188704139–188,926,054	1.9 × 10^−4^
rs81237965	3	3,693,136	intron	*RADIL*	3: 3618093–313,033	1.9 × 10^−4^
rs81344023	6	153,022,279	intron	*FGGY*	6: 152754730–153,213,125	2.1 × 10^−4^
rs81349902	1	188,903,236	intron	*RTN1*	1: 188704139–188,926,054	2.3 × 10^−4^
rs318950111	3	43,561,730	downstream	*ZNF169*	3: 43513397–43,558,613	2.5 × 10^−4^
rs81452423	15	32,935,177	intergenic	*DLGAP2*	15: 33173120–33,264,155	3.0 × 10^−4^

^a^SSC = *Sus scrofa* chromosome

^b^Location in Ensembl Sscrofa11.1

^c^If the variant was intergenic, the closest gene within a 1 Mbp window was indicated

^d^SNVs under the suggestive significance threshold of 1.0 × 10^−5^ in bold [39]

**Table 3:** Top 15 SNVs ranked by significance for the GWAS analysis of isolation of *Salmonella* from tissues at slaughter

SNV ID	SSC^**a**^	Location (bp)^**b**^	Variant type	Gene^**c**^	Gene location^**b**^	***p***-value^**d**^
rs322440805	3	130,637,256	intergenic	*DCDC2C*	3: 131097730–131,194,726	**1.6 × 10**^**−6**^
rs326411709	3	130,676,894	intergenic	*DCDC2C*	3: 131097730–131,194,726	**3.7 × 10**^**− 6**^
rs319944764	3	130,689,632	intergenic	*DCDC2C*	3: 131097730–131,194,726	**8.6 × 10**^**−6**^
rs81348815	1	15,081,855	intergenic	*AKAP12*	1: 14906823–15,016,841	2.0 × 10^−5^
rs80951933	1	15,109,785	intergenic	*AKAP12*	1: 14906823–15,016,841	3.1 × 10^−5^
rs80903645	1	15,064,589	intergenic	*AKAP12*	1: 14906823–15,016,841	3.6 × 10^−5^
rs326617356	18	26,691,633	intergenic	*KCND2*	18: 26554190–26,623,034	1.3 × 10^− 4^
rs323563819	19	109,560,455	intron	*GPC3*	X: 109536447–110,060,245	2.0 × 10^−4^
rs80820138	14	122,383,711	intergenic	*GPAM*	14: 122542205–122,577,239	2.0 × 10^−4^
rs80961723	7	120,400,325	intergenic	*SETD3*	7: 120412549–120,486,627	2.2 × 10^−4^
rs320610499	6	141,349,179	intron	*NEGR1*	6: 141115729–141,647,820	2.5 × 10^−4^
rs81296290	2	91,504,717	intron	*VCAN*	2: 91287931–91,811,674	2.8 × 10^−4^
rs324243793	18	38,747,572	intron	*DPY19L2*	18: 38693003–38,764,024	3.3 × 10^−4^
rs80840697	1	14,988,130	intron	*AKAP12*	1: 14906823–15,016,841	4.1 × 10^−4^
rs81361262	2	90,665,766	downstream	*ATG10*	2: 90437020–90,665,380	4.5 × 10^−4^

^a^SSC = *Sus scrofa* chromosome

^b^Location in Ensembl Sscrofa11.1

^c^If the variant was intergenic, the closest gene within a 1 Mbp window was indicated

^d^SNVs under the suggestive significance threshold of 1.0 × 10^−5^ in bold [39]

**Table 4:** Top 15 SNVs ranked by significance for the GWAS analysis of *Salmonella* seropositivity from end of nursery to end of finisher

SNV ID	SSC^**a**^	Location (bp)^**b**^	Variant type	Gene^**c**^	Gene location^**b**^	***p***-value^**d**^
rs81241392	17	28,616,449	intron	*RALGAPA2*	17: 28394827–28,679,538	7.6 × 10^− 5^
rs323410857	1	201,886,813	intron	*HACD4*	1: 201886332–201,910,664	8.2 × 10^− 5^
rs81459294	16	1,133,302	intron	*CTNND2*	16: 508245–1,521,550	1.0 × 10^− 4^
rs80868434	7	30,048,501	intergenic	*MLN*	7: 30003291–30,013,128	1.2 × 10^− 4^
rs80849858	1	77,472,299	intergenic	*TRAF3IP2*	1: 77484191–77,523,887	1.2 × 10^− 4^
rs336677749	17	28,600,733	intron	*RALGAPA2*	17: 28394827–28,679,538	1.3 × 10^− 4^
rs330020208	6	89,629,789	intron	*PHC2*	6: 89574035–89,685,278	1.3 × 10^− 4^
rs81370878	3	56,211,114	intergenic	*VWA3B*	3: 55950591–56,171,826	1.4 × 10^− 4^
rs323186575	6	91,486,462	intron	*ZMYM4*	6: 91455778–91,623,818	1.4 × 10^−4^
rs80970182	17	28,210,436	intron	*CFAP61*	17: 28111660–28,372,543	1.4 × 10^−4^
rs80930168	1	77,338,889	intron	*REV3L*	1: 77195253–77,401,051	1.9 × 10^−4^
rs80801203	17	28,465,351	intron	*RALGAPA2*	17: 28394827–28,679,538	2.1 × 10^−4^
rs338087144	14	77,361,157	intergenic	*KAT6B*	14: 77404443–77,597,673	2.4 × 10^−4^
rs81241392	17	28,616,449	intron	*RALGAPA2*	17: 28394827–28,679,538	2.6 × 10^−4^
rs81290595	15	6,253,250	intron	*ENSSSCG00000036561*	15: 6222423–6,266,771	2.8 × 10^−4^

^a^SSC = *Sus scrofa* chromosome

^b^Location in Ensembl Sscrofa11.1

^c^If the variant was intergenic, the closest gene within a 1 Mbp window was indicated

^d^SNVs under the suggestive significance threshold of 1.0 × 10^−5^ in bold [39]

## Discussion

This study aimed to assess susceptibility to *Salmonella* in commercial pigs by using a GWAS approach to identify potential variants associated with *Salmonella* IgG antibody response from end of nursery to end of finisher, *Salmonella* shedding from weaning to end of finisher, and isolation of *Salmonella* from tissues at slaughter. GWAS is a useful tool in preliminary identification of novel SNVs or genes and associations between genetic variants and disease resistance or susceptibility. This has promise for future efforts in breeding for resistance to *Salmonella* on-farm and at slaughter, which may lead to a reduction of *Salmonella* benefiting both public health and animal welfare.

### GWAS analysis of shedding from nursery to finisher stage

GWAS analysis identified two associated SNVs. The first was an intron variant located in the *PTPRJ* gene, which encodes protein tyrosine phosphatase (PTP) receptor type J (PTPRJ) and was observed at a higher frequency in pigs that never shed *Salmonella*. These PTPRJ receptors are found on many cell types and are highly expressed in the macrophage rich tissues of the intestines [40]. PTPRJ receptors are responsible for negative regulation of protein tyrosine kinases, which are involved in pathogen recognition and clearance, as well as resolution of inflammation [41–44]. PTPs may also be involved in B cell activation, recruitment of neutrophils, and the negative regulation of T-cell signaling [39, 45–47]. The timely recruitment of neutrophils is a crucial part of the immune response and often plays a key role in the clearance of pathogens in the gut [48]. A challenge study on *PTPRJ*-deficient mice showed impairment of B cell development but an unexpected significant increase in early neutrophil recruitment and rapid clearance of *Staphylococcus aureus* in a subcutaneous air pouch and suggested that PTPRJ plays both a positive and negative role in attraction of neutrophils [49].

Interestingly, the *PTPRJ* variant was observed at a significantly higher frequency in pigs with no *Salmonella* shedding observed from weaning to end of finisher. It is possible that, this variant may be causing a reduction in the expression of *PTPRJ* resulting in a subsequent rapid increase in neutrophil recruitment and clearance of *Salmonella* infection in the control population [49], while RPTP, another member of the PTP family, is rescuing some B cell functionality and signalling [48]. Currently, more research is needed into the putative function of PTPRJ in pigs and how this variant and its interactions with other PTPs, like RPTP, may affect B cell signalling and neutrophil recruitment in response to pathogens like *Salmonella* before a definitive conclusion can be made.

Analysis of the shedding trait also identified an intergenic variant nearest to the *ST6GALNAC3* gene, which encodes ST6 (α-N-acetylneuraminyl-2,-3-β-galactosyl-1,3)-N-acetylgalactosamine-α-2,6-sialyltransferase 3 (ST6GALNAC3) and was also more frequent in pigs that did not shed *Salmonella*. ST6GALNAC3 is a member of a family of sialylatransferases involved in the transfer of sialic acids CMP-sialic acid to O-glycans with α-2,6 linkage [50–52]. Sialylated glycans, like those produced by ST6GALNAC3, are involved in many key cellular processes including cellular adhesion, self-recognition, and signaling [51, 53–55]. Currently, little is known about the in vivo function and differential expression of *ST6GALNAC3* in pigs [52, 56]. However, in vitro studies have demonstrated that sialic acid moieties on the mucosal surface may be used by pathogens, including *Salmonella*, as a nutrient source or as receptors for adhesion and invasion [51, 55, 57, 58]. One such study found that removal of N-acetylneuraminic acid, the most abundant cell surface sialic acid, greatly impaired *Salmonella* attachment to Caco-2 cells [57]. Additionally GD1a, synthesized by ST6GALNAC3, has also been implicated as a co-receptor for *Salmonella* flagellin (FliC) and is thought to induce β-defensin-2 in Caco-2 cells [59].

To date, the function and expression of *ST6GALNAC3* largely remains an unknown in pigs. In the current study, the variant was seen at a higher frequency in pigs that were not observed to be shedding from weaning to finisher stage. Due to the role of sialic acids in *Salmonella* adhesion and nutrient acquisition, it is possible that this variant near *ST6GALNAC3* is resulting in a change of the sialylation of the mucosal surface. This may be impacting either the availability and localization of host-derived sialylated glycans and impairing the rate of *Salmonella* adherence or nutrient acquisition and thus may affect *Salmonella* susceptibility in pigs.

### GWAS analysis of colonization at slaughter

The model for isolation of *Salmonella* from tissues at slaughter identified three intergenic variants with a suggestive association nearest to the gene encoding doublecortin domain containing 2C (DCDC2C). Two of these variants were more frequent in pigs that tested negative at slaughter, while the third was seen at a higher frequency in pigs positive at slaughter. The *DCDC2C* gene is currently uncharacterized in pigs and little is known about its function in vivo. Studies of *DCDC2C* in humans have suggested that variants in this gene may be associated with structural defects in cilia in sperm and in cilia length in sensory cells in the ear [60, 61]. Thus, it is possible that the variants observed in the current study also result in structural defects, either in the cilia in the lungs, or perhaps the villi of the intestines of pigs and may be changing the pigs’ susceptibility to *Salmonella.* How tissue colonization at slaughter may be being altered is yet unclear, as SNVs associated with both negative and positive isolation of *Salmonella* at slaughter were identified. Additionally, these variants are approximately 400 kbp from the *DCDC2C* gene, and thus the observed effect on the slaughter trait may be related, in part, to linkage with a functional SNV in a different gene. Regardless, DCDC2C may be a potential target for further investigation *Salmonella* susceptibility in pigs.

Analysis of the slaughter model identified an additional three intergenic variants near the gene encoding A-kinase anchor protein 12 (AKAP12) that were approaching the suggestive association threshold. An intron variant in *AKAP12* was also identified in the top 15 significant SNVs for the slaughter trait. AKAP12 is a member of the AKAP family of scaffolding proteins involved in the recruitment and anchoring of protein kinases (PK) A and B. AKAP12 has be shown to regulate the subcellular localization of PKA and play a role in PKB regulation [62, 63]. PKA and PKB signaling play a crucial role in intramacrophage killing. *Salmonella* is known to subvert the host cell machinery and manipulate these pathways to persist intracellularly through the reduction of reactive oxygen intermediates or the manipulation of motor proteins [64, 65]. Studies have shown that inhibiting PKA and PKB activation greatly reduces virulence and impairs intracellular growth and survival of *Salmonella* [66, 67].

In light of the role that AKAP12 plays in the localization and regulation of PKA and PKB, which are key players in the intracellular survival of *Salmonella*, it is possible that variants near *AKAP12* are altering the expression of *AKAP12* and reducing or inhibiting the normal localization of PKA and PKB. This, in turn, may be impairing intracellular survival of *Salmonella* in host macrophages and preventing or limiting the systemic spread of *Salmonella* to lymphatic tissues and may account for the significantly higher frequency of these variants in the control pigs that were negative for *Salmonella* in tissues collected at slaughter.

### GWAS analysis of seropositivity from nursery to finisher stage

GWAS analysis did not identify any significant associations with the tested variants. The closest SNV approaching the suggestive association threshold was an intergenic variant nearest to the gene *RALGAPA2*, which encodes the gene Ral GTPase-activating protein (RalGAP) catalytic alpha subunit 2 (α2) of RalGAP and was observed at a higher frequency in pigs that tested seropositive for *Salmonella* at least once. An additional two intergenic variants near and one intron variant in *RALGAPA2* were identified in the top 15 significant SNVs for the seropositivity model. RalGAPs are involved in the negative regulation of RalGTPases, RalA and RalB, in the Ras/Ral signaling pathways and regulate crucial cellular processes including response to infection and mediation of inflammation [68–72]. Notably, constitutive expression of RalGTPases has been shown to promote tumorigenesis, increased expression of inflammatory cytokines, and increased epithelial permeability [69, 73–76].

It is possible that these variants are altering the expression of *RALGAPA2* and, in turn, causing constitutive expression of RalGTPases. Considering the role of Ral GTPases in cell survival, inflammation, and permeability, this constitutive expression may be promoting susceptibility to *Salmonella*. Firstly, by creating an inflammatory environment in which *Salmonella* is known to thrive [77–79], or secondly, by impairing infected cell death and promoting the intracellular survival of *Salmonella* leading to chronic or persistent infection and higher antibody production [80–83]. However, it is important to note that the ELISA chosen for the current study tested for *Salmonella* IgG antibodies, but it is possible that some pigs had only produced a preliminary IgM response. Further studies are needed to assess the presence of IgM antibodies and whether they may result in stronger associations between the identified variants and *Salmonella* seropositivity. Additionally, a targeted study into *RALGAPA2* and its pathways may identify an association with *Salmonella* susceptibility that was missed at the genome-wide level.

## Conclusion

To the best of the authors knowledge, this is the first GWAS analysis assessing susceptibility to *Salmonella* in pigs at different stage of production on commercial farms and at slaughter. The variants identified herein were generally in or near genes involved in broad innate immunity and may be of great interest in improving genetic resistance to control enteric pathogens on swine farms. Overall, much of the porcine innate and humoral immune responses to these pathogens remains unknown. Further investigation of these genes on-farm, and the function, expression, and pathways of these genes both in vitro may greatly improve our understanding of the genetic basis of susceptibility to *Salmonella* and other swine enteric pathogens.

## Methods

Animal use in this project was approved by the University of Guelph Animal Care Committee (AUP# 3124) and follows Canadian Council of Animal Care guidelines (CCAC, 2009).

### Animals and sample collection

Farm and pig selection for this study have been detailed previously [84]. Briefly, 14 groups of 54–60 pigs (three-way Yorkshire x Landrace x terminal boar line cross) were selected from eight commercial farrowing sources in Southern Ontario; all pigs were housed on commercial farms and kept under the management of the farm personnel. Two groups of pigs were selected from each of six farrowing sources (designated Cohorts One and Two), while the remaining two farrowing sources had only one group each (Cohort One) for a total of 14 groups. During the nursery stage, pigs received either a conventional diet (high complexity) or a lower cost reduced animal protein diet (low complexity) in which animal protein was replaced by plant protein as part of a larger study [85]. After which, all pigs received conventional diets during grower-finisher stage.

Blood and fecal samples were collected at weaning, and at the end of the nursery, grower and finisher stages. Rectal swabs (Starplex®, VWR International, Mississauga, Ontario, Canada) were taken in the event that a fecal sample could not be obtained. Blood was drawn from the jugular or suborbital vein, transported to the lab in a cooler, and then centrifuged at 1500 x g for 20 min. Sera were stored at − 20 °C. At the end of production pigs were shipped to the abattoir for slaughter where palatine tonsils and submandibular lymph nodes were collected from a subset of 580 pigs.

### *Salmonella* antibody detection

Collected sera were tested for IgG antibodies to *Salmonella* O-antigens 1, 3, 4, 6, 7, 9, 10, and 12 via a commercial enzyme-linked immunosorbent assay (ELISA; pigtype® *Salmonella* Ab kit, QIAGEN Leipzig GmbH, Leipzig, Germany) as described previously [86]. Sample optical density (OD) was measured at 450 nm using a BioTek Synergy HT Multi-Mode Microplate Reader and BioTek’s Gen5 software version 11.1. The sample-to-positive (S/P) ratios were calculated as follows:

$$ \mathrm{S}/\mathrm{P}=\frac{{\mathrm{OD}}_{\mathrm{sample}}-\mathrm{Mean}\ {\mathrm{OD}}_{\mathrm{negative}\ \mathrm{control}}}{\mathrm{Mean}\ {\mathrm{OD}}_{\mathrm{positive}\ \mathrm{control}}-\mathrm{Mean}\ {\mathrm{OD}}_{\mathrm{negative}\ \mathrm{control}}} $$

A sample was considered seropositive if the S/P ratio was greater or equal to 0.3.

### *Salmonella* isolation

Fecal samples and tissues were cultured as detailed previously [84]. Briefly, 10 g of fecal or tissue sample was homogenized in 50 mL of tetrathionate broth (Oxoid, Nepean, Ontario, Canada) using a Seward Stomacher 400 Circulator (Seward Laboratory Systems Inc., Bohemia, New York, USA) and incubated for 24 h at 37 °C. After which, 0.1 mL of tetrathionate broth culture was transferred into 9.9 mL Rappaport Vassiliadis (RV) broth (Oxoid, Nepean, Ontario, Canada) followed by another 24 h incubation at 42 °C. Then, a loopful of RV culture was plated onto xylose-lysine-tergitol 4 agar (Becton Dickinson™, Baltimore, Maryland, USA) and incubated for a final 24 h at 37 °C. *Salmonella* colonies were confirmed by a *Salmonella* O Antiserum Poly A-I and Vi (Becton Dickinson™, Grayson, Georgia, USA) slide agglutination test.

### *Salmonella* phenotypes

The following binary traits were defined for each pig: *Salmonella* seropositivity from end of nursery to end of finisher (never seropositive vs. seropositive once or more), *Salmonella* shedding from weaning to end of finisher (never shed vs. shed once or more), and positive isolation of *Salmonella* from tonsils and/or lymph nodes at slaughter (yes/no). Seropositivity at weaning was excluded when categorizing pigs into binary traits due to the concern of maternal antibodies confounding the results in the GWAS. Supplementary Figure 1 depicts a simplified flow chart of the sample collection and case-control groups by phenotype.

### Genotyping and quality control

DNA was extracted from tail dockings, ear tissue, or blood using the DNEasy Blood and Tissue kit (Qiagen, Mississauga, Ontario Canada), with either 25 mg of tissue or 100 ul of blood [35]. DNA was genotyped at Eurofins BioDiagnostics, Inc. (River Falls, Wisconsin, USA) using a custom 54 K Affymetrix Axiom® myDesign™ chip designed in consultation with the Canadian Centre for Swine Improvement (Ottawa, Ontario, Canada). Quality control (QC) of SNVs was performed in PLINK v1.9 [87, 88]. As this chip contained select SNVs from multiple research groups, some of which had proprietary labels that precluded their identification in the genome, SNVs were removed if they had no corresponding rsID in Ensembl Sscrofa11.1. Pigs with SNV call rates of less than 90% were excluded and SNVs with a minor allele frequency lower than 5% or a call rate less than 95% were removed from further analysis. The SNV call rates were compared between cases and controls, and Fisher’s exact test was used to exclude any SNV in which the missingness between a case and control was significantly different (*p* < 1.0 × 10^− 5^).

### Genome-wide association study

A GWAS model was used to assess each of the phenotypes described above. Pigs that tested seropositive for *Salmonella* or shed *Salmonella* at least once on-farm, and pigs that were positive for isolation of *Salmonella* at slaughter were considered to be the case population. Pigs that never tested seropositive, shed *Salmonella*, or tested negative at slaughter were assigned as the control population for the GWAS models. The outcome variable for each model was seropositive at least once from end of nursery to end of finisher (seropositivity model), shedding detected at least once from weaning to end of finisher (shedding model), and positive isolation at slaughter (colonization model), respectively.

Univariable analysis of each phenotype was performed using a mixed-effects logistic regression model in Stata (Stata/IC 14.2 for Windows, StataCorp LP, Texas, USA) to assess the significance of associations between covariates and the phenotype (*p* < 0.05) and subsequent inclusion in the GWAS model [84, 86]. For the seropositivity model, only farm was included as a covariate. Covariates included in the shedding model were diet and cohort, while covariates for the colonization model were age at slaughter, cohort, and season. To account for the cryptic relatedness of the pigs in a study population with no sire information, a genomic relatedness matrix (GRM) was calculated using the genome-wide efficient mixed model association algorithm (GEMMA) v0.96 [37, 38, 89]. Genome-wide analyses of case-control data were performed using a generalized logistic mixed model association test (GMMAT) [90] using the GMMAT v0.9.3 package for R [91, 92]. The covariates analyzed in Stata and the GRM created in GEMMA were included in the GMMAT analysis. The chosen *p*-value threshold for suggestive and significant associations was 1.0 × 10^− 5^ and 5.0 × 10^− 7^, respectively based on a study by Burton et al. [39]. The Wald test was used to assess the significance of associations between SNVs and the *Salmonella* phenotypes. Allele frequencies in case-control populations were calculated and an allelic chi-squared (χ^2^) test in PLINK v1.9 was used to determine if there was a significant difference between case and control pig populations. In the event that an intergenic variant was identified as significant, the closest gene within a 1 Mbp window, either upstream or downstream, was assumed to be the causative gene. The data generated from this GWAS has been deposited in the Animal Quantitative Trait Loci Database (AnimalQTLdb) and can be accessed as a group via https://www.animalgenome.org/QTLdb/supp/?t=MwAn1O1XiA, or individually via association (QTL) numbers: 194531–194535.

## Supplementary information

**Additional file 1: Figure S1.** Visual representation of the sampling and processing of sera and fecal/tissue samples from pigs on-farm and at slaughter and the case-control population breakdowns of the three binary *Salmonella* phenotypes used in GWAS.

**Additional file 2: Table S1.** Allele frequencies in case and control pig populations for SNVs with significant associations in GWAS models.

## Data Availability

The data generated from this GWAS has been deposited in the Animal Quantitative Trait Loci Database (AnimalQTLdb) and can be accessed as a group via https://www.animalgenome.org/QTLdb/supp/?t=MwAn1O1XiA, or individually via association (QTL) numbers: 194531-194535.

## Abbreviations

AKAP: A-kinase anchoring protein
ELISA: Enzyme-linked immunosorbent assay
DCDC2C: Doublecortin domain containing 2C
GWAS: Genome-wide association study
GRM: Genomic relatedness matrix
GMMAT: Generalized logistic mixed model association test
GEMMA: Genome-wide efficient mixed model association
IgG: Immunoglobulin G
MBL: Mannan-binding lectin
NOD: Nucleotide-binding oligomerization domain-containing protein
OD: Optical density
PK: Protein kinase
PTP: Protein tyrosine phosphatase
PTPRJ: Protein tyrosine phosphatase receptor type J
QC: Quality control
RalGAP: Ral GTPase-activating protein
RALGAPA2: Ral GTPase-activating protein catalytic alpha subunit 2
RV: Rappaport Vassiliadis
*S*.: *Salmonella*
SNV: Single-nucleotide variant
S/P: Sample-to-positive
ST6GALNAC3: ST6 (α-N-acetylneuraminyl-2,-3-β-galactosyl-1,3)-N-acetylgalactosamine-α-2,6-sialyltransferase 3
TLR: Toll-like receptor

## References

[1] Thomas MK, Murray R, Flockhart L, Pintar K, Pollari F, Fazil A (2013). Estimates of the burden of foodborne illness in Canada for 30 specified pathogens and unspecified agents, circa 2006. Foodborne Pathog Dis.

[2] Pires SM, Vieira AR, Hald T, Cole D (2014). Source attribution of human salmonellosis: an overview of methods and estimates. Foodborne Pathog Dis.

[3] Majowicz SE, Musto J, Scallan E, Angulo FJ, Kirk M, O’Brien SJ (2010). The global burden of nontyphoidal Salmonella gastroenteritis. Clin Infect Dis.

[4] European Food Safety Authority (2015). The European Union summary report on trends and sources of zoonoses, zoonotic agents and food-borne outbreaks in 2014. EFSA J.

[5] Jackson BR, Griffin PM, Cole D, Walsh KA, Chai SJ (2013). Outbreak-associated *Salmonella enterica* serotypes and food commodities, United States, 1998–2008. Emerg Infect Dis.

[6] Nesbitt A, Ravel A, Murray R, McCormick R, Savelli C, Finley R (2012). Integrated surveillance and potential sources of *Salmonella enteritidis* in human cases in Canada from 2003 to 2009. Epidemiol Infect.

[7] Ravel A, Greig J, Tinga C, Todd E, Campbell G, Cassidy M (2009). Exploring historical Canadian foodborne outbreak data sets for human illness attribution. J Food Prot.

[8] Bolton DJ, Ivory C, McDowell D (2013). A study of Salmonella in pigs from birth to carcass: Serotypes, genotypes, antibiotic resistance and virulence profiles. Int J Food Microbiol.

[9] Farzan A, Friendship RM, Dewey CE, Muckle AC, Gray JT, Funk J (2008). Distribution of *Salmonella* serovars and phage types on 80 Ontario swine farms in 2004. Can J Vet Res.

[10] Merialdi G, Barigazzi G, Bonilauri P, Tittarelli C, Bonci M, Dottori M (2008). Longitudinal study of *Salmonella* infection in Italian farrow-to-finish swine herds. Zoonoses Public Health.

[11] Funk JA, Davies PR, Nichols MA (2001). Longitudinal study of *Salmonella enterica* in growing pigs reared in multiple-site swine production systems. Vet Microbiol.

[12] Vigo GB, Cappuccio JA, Piñeyro PE, Salve A, Machuca MA, Quiroga MA (2009). *Salmonella enterica* subclinical infection: bacteriological, serological, pulsed-field gel electrophoresis, and antimicrobial resistance profiles--longitudinal study in a three-site farrow-to-finish farm. Foodborne Pathog Dis.

[13] Boyen F, Haesebrouck F, Maes D, Van Immerseel F, Ducatelle R, Pasmans F (2008). Non-typhoidal *Salmonella* infections in pigs: A closer look at epidemiology, pathogenesis and control. Vet Microbiol.

[14] Flockhart L, Pintar K, Cook A, McEwen S, Friendship R, Kelton D (2017). Distribution of *Salmonella* in Humans, Production Animal Operations and a Watershed in a FoodNet Canada Sentinel Site. Zoonoses Public Health.

[15] Deckert A, Gow S, Rosengren L, Léger D, Avery B, Daignault D (2010). Canadian integrated program for antimicrobial resistance surveillance (CIPARS) farm program: Results from finisher pig surveillance. Zoonoses Public Health.

[16] Bearson BL, Bearson SMD (2011). Host specific differences alter the requirement for certain *Salmonella* genes during swine colonization. Vet Microbiol.

[17] Zhao S, Zhu M, Chen H (2012). Immunogenomics for identification of disease resistance genes in pigs: a review focusing on Gram-negative bacilli. J Anim Sci Biotechnol.

[18] Hotes S, Traulsen I, Krieter J (2011). *Salmonella* control measures with special focus on vaccination and logistic slaughter procedures. Transbound Emerg Dis.

[19] Davies G, Genini S, Bishop SC, Giuffra E (2009). An assessment of opportunities to dissect host genetic variation in resistance to infectious diseases in livestock. Animal.

[20] Doyle MP, Erickson MC (2006). Reducing the Carriage of foodborne pathogens in livestock and poultry. Poult Sci.

[21] Picard B, Lebret B, Cassar-Malek I, Liaubet L, Berri C, Le Bihan-Duval E (2015). Recent advances in omic technologies for meat quality management. Meat Sci.

[22] Miar Y, Plastow G, Bruce H, Moore S, Manafiazar G, Kemp R (2014). Genetic and phenotypic correlations between performance traits with meat quality and carcass characteristics in commercial crossbred pigs. PLoS One.

[23] Knap PW, Rauw WM. Selection for high production in pigs. In: Resource Allocation Theory Applied to Farm Animal Production. Reno, Nevada, USA: CAB International; 2008. p. 210–229. [cited 2019 Apr 22] Available from: https://www-cabi-org.subzero.lib.uoguelph.ca/cabebooks/FullTextPDF/2009/20093002860.pdf.

[24] Mallard BA, Wilkie BN, Kennedy BW, Quinton M (1992). Use of estimated breeding values in a selection index to breed Yorkshire pigs for high and low immune and innate resistance factors. Anim Biotechnol.

[25] Clapperton M, Diack AB, Matika O, Glass EJ, Gladney CD, Mellencamp MA (2009). Traits associated with innate and adaptive immunity in pigs: heritability and associations with performance under different health status conditions. Genet Sel Evol.

[26] Skjolaas KA, Burkey TE, Dritz SS, Minton JE (2006). Effects of *Salmonella enterica* serovars Typhimurium (ST) and Choleraesuis (SC) on chemokine and cytokine expression in swine ileum and jejunal epithelial cells. Vet Immunol Immunopathol.

[27] Crawley AM, Mallard B, Wilkie BN (2005). Genetic selection for high and low immune response in pigs: effects on immunoglobulin isotype expression. Vet Immunol Immunopathol.

[28] van Diemen P, Kreukniet M, Galina L, Bumstead N, Wallis T (2002). Characterisation of a resource population of pigs screened for resistance to salmonellosis. Vet Immunol Immunopathol.

[29] Edfors-Lilja I, Wattrang E, Magnusson U, Fossum C (1994). Genetic variation in parameters reflecting immune competence of swine. Vet Immunol Immunopathol.

[30] Buschmann H, Herrmann H, Meyer J, Kleinschmidt A (1985). Quantitative immunological parameters in pigs-experiences with the evaluation of an immunocompetence profile. Z Tierz Züchtungsbiol.

[31] Kich JD, Uthe JJ, Benavides MV, Cantão ME, Zanella R, Tuggle CK (2014). TLR4 single nucleotide polymorphisms (SNPs) associated with *Salmonella* shedding in pigs. J Appl Genet.

[32] Shinkai H, Suzuki R, Akiba M, Okumura N, Uenishi H (2011). Porcine Toll-like receptors: recognition of *Salmonella enterica* serovar Choleraesuis and influence of polymorphisms. Mol Immunol.

[33] Burkey TE, Skjolaas KA, Dritz SS, Minton JE (2007). Expression of Toll-like receptors, interleukin 8, macrophage migration inhibitory factor, and osteopontin in tissues from pigs challenged with *Salmonella enterica* serovar Typhimurium or serovar Choleraesuis. Vet Immunol Immunopathol.

[34] Keirstead ND, Hayes MA, Vandervoort GE, Brooks AS, Squires EJ, Lillie BN (2011). Single nucleotide polymorphisms in collagenous lectins and other innate immune genes in pigs with common infectious diseases. Vet Immunol Immunopathol.

[35] Ainslie-Garcia MH, Farzan A, Jafarikia M, Lillie BN (2018). Single nucleotide variants in innate immune genes associated with *Salmonella* shedding and colonization in swine on commercial farms. Vet Microbiol.

[36] Amos W, Driscoll E, Hoffman JI (2011). Candidate genes versus genome-wide associations: which are better for detecting genetic susceptibility to infectious disease?. Proceedings Biol Sci.

[37] Zhou X, Stephens M (2012). Genome-wide efficient mixed-model analysis for association studies. Nat Genet.

[38] Voight BF, Pritchard JK (2005). Confounding from cryptic relatedness in case-control association studies. PLoS Genet.

[39] Burton PR, Clayton DG, Cardon LR, Craddock N, Deloukas P, Duncanson A (2007). Genome-wide association study of 14,000 cases of seven common diseases and 3,000 shared controls. Nature.

[40] Dave RK, Dinger ME, Andrew M, Askarian-Amiri M, Hume DA, Kellie S (2013). Regulated expression of PTPRJ/CD148 and an antisense long noncoding RNA in macrophages by proinflammatory stimuli. PLoS One.

[41] Hirayama D, Iida T, Nakase H. The phagocytic function of macrophage-enforcing innate immunity and tissue homeostasis. Int J Mol Sci. 2017;19(1) [cited 2018 Nov 2]Available from: http://www.ncbi.nlm.nih.gov/pubmed/29286292. 10.3390/ijms19010092 PMC5796042 29286292

[42] Gordon S (2012). Innate immune functions of macrophages in different tissue environments. J Innate Immun.

[43] Weiss A (2009). Kinases and phosphatases of the immune system. Immunol Rev.

[44] Shanley TP (2002). Phosphatases: Counterregulatory role in inflammatory cell signaling. Crit Care Med.

[45] Lin J, Zhu JW, Baker JE, Weiss A (2004). Regulated expression of the receptor-like tyrosine phosphatase CD148 on hemopoietic cells. J Immunol.

[46] Lin J, Weiss A (2003). The tyrosine phosphatase CD148 is excluded from the immunologic synapse and down-regulates prolonged T cell signaling. J Cell Biol.

[47] Baker JE, Majeti R, Tangye SG, Weiss A (2001). Protein tyrosine phosphatase CD148-mediated inhibition of T-cell receptor signal transduction is associated with reduced LAT and phospholipase Cgamma1 phosphorylation. Mol Cell Biol.

[48] Zhu JW, Doan K, Park J, Chau AH, Zhang H, Lowell CA (2011). Receptor-like tyrosine phosphatases CD45 and CD148 have distinct functions in chemoattractant-mediated neutrophil migration and response to *S. aureus*. Immunity.

[49] Zhu JW, Brdicka T, Katsumoto TR, Lin J, Weiss A (2008). Structurally distinct phosphatases CD45 and CD148 both regulate B cell and macrophage immunoreceptor signaling. Immunity.

[50] Wang L, Liu Y, Wu L, Sun X-L (2016). Sialyltransferase inhibition and recent advances. Biochim Biophys Acta - Proteins Proteomics.

[51] Huang Y-L, Chassard C, Hausmann M, von Itzstein M, Hennet T (2015). Sialic acid catabolism drives intestinal inflammation and microbial dysbiosis in mice. Nat Commun.

[52] Takashima S, Tsuji S. Handbook of glycosyltransferases and related genes. 2nd ed. Japan: Springer; c2014. p. 737–47. [cited 2018 Nov 3] Available from: https://www.springer.com/la/book/9784431542391#aboutBook.

[53] Kim J-W, Park H-J, Chae S-K, Ahn J-H, G-Y DO, Choo Y-K (2016). Ganglioside GD1a promotes oocyte maturation, furthers preimplantation development, and increases blastocyst quality in pigs. J Reprod Dev.

[54] Tsuchida A, Ogiso M, Nakamura Y, Kiso M, Furukawa K, Furukawa K (2005). Molecular cloning and expression of human ST6GalNAc III: restricted tissue distribution and substrate specificity. J Biochem.

[55] Vimr ER, Kalivoda KA, Deszo EL, Steenbergen SM (2004). Diversity of microbial sialic acid metabolism. Microbiol Mol Biol Rev.

[56] Sjoberg ER, Kitagawa H, Glushka J, van Halbeek H, Paulson JC (1996). Molecular cloning of a developmentally regulated N-acetylgalactosamine alpha2,6-sialyltransferase specific for sialylated glycoconjugates. J Biol Chem.

[57] Arabyan N, Park D, Foutouhi S, Weis AM, Huang BC, Williams CC (2016). *Salmonella* degrades the host glycocalyx leading to altered infection and glycan remodeling. Sci Rep.

[58] Ng KM, Ferreyra JA, Higginbottom SK, Lynch JB, Kashyap PC, Gopinath S (2013). Microbiota-liberated host sugars facilitate post-antibiotic expansion of enteric pathogens. Nature.

[59] Ogushi K, Wada A, Niidome T, Okuda T, Llanes R, Nakayama M (2004). Gangliosides act as co-receptors for *Salmonella enteritidis* FliC and promote FliC induction of human beta-defensin-2 expression in Caco-2 cells. J Biol Chem.

[60] Jumeau F, Chalmel F, Fernandez-Gomez FJ, Carpentier C, Obriot H, Tardivel M (2017). Defining the human sperm microtubulome: An integrated genomics approach. Biol Reprod.

[61] Grati M, Chakchouk I, Ma Q, Bensaid M, Desmidt A, Turki N (2015). A missense mutation in DCDC2 causes human recessive deafness DFNB66, likely by interfering with sensory hair cell and supporting cell cilia length regulation. Hum Mol Genet.

[62] Gelman IH (2010). Emerging roles for SSeCKS/Gravin/AKAP12 in the control of cell proliferation, cancer malignancy, and barriergenesis. Genes Cancer.

[63] Akakura S, Huang C, Nelson PJ, Foster B, Gelman IH (2008). Loss of the SSeCKS/Gravin/AKAP12 gene results in prostatic hyperplasia. Cancer Res.

[64] Kashina AS, Semenova IV, Ivanov PA, Potekhina ES, Zaliapin I, Rodionov VI (2004). Protein kinase A, which regulates intracellular transport, forms complexes with molecular motors on organelles. Curr Biol.

[65] Gallois A, Klein JR, Allen LA, Jones BD, Nauseef WM (2001). *Salmonella* pathogenicity island 2-encoded type III secretion system mediates exclusion of NADPH oxidase assembly from the phagosomal membrane. J Immunol.

[66] Kuijl C, Savage NDL, Marsman M, Tuin AW, Janssen L, Egan DA (2007). Intracellular bacterial growth is controlled by a kinase network around PKB/AKT1. Nature.

[67] Uchiya K, Nikai T (2004). *Salmonella enterica* serovar Typhimurium infection induces cyclooxygenase 2 expression in macrophages: involvement of *Salmonella* pathogenicity island 2. Infect Immun.

[68] Moghadam AR, Patrad E, Tafsiri E, Peng W, Fangman B, Pluard TJ (2017). Ral signaling pathway in health and cancer. Cancer Med.

[69] Bodemann BO, Orvedahl A, Cheng T, Ram RR, Ou Y-H, Formstecher E (2011). RalB and the exocyst mediate the cellular starvation response by direct activation of autophagosome assembly. Cell.

[70] Cox AD, Der CJ (2010). Ras history. Small GTPases.

[71] Shirakawa R, Fukai S, Kawato M, Higashi T, Kondo H, Ikeda T (2009). Tuberous sclerosis tumor suppressor complex-like complexes act as GTPase-activating proteins for Ral GTPases. J Biol Chem.

[72] Feig LA (2003). Ral-GTPases: approaching their 15 minutes of fame. Trends Cell Biol.

[73] Wang XQ, Sun P, Paller AS (2001). Inhibition of integrin-linked kinase/protein kinase B/Akt signaling: mechanism for ganglioside-induced apoptosis. J Biol Chem.

[74] Peschard P, McCarthy A, Leblanc-Dominguez V, Yeo M, Guichard S, Stamp G (2012). Genetic deletion of RALA and RALB small GTPases reveals redundant functions in development and tumorigenesis. Curr Biol.

[75] Saito R, Shirakawa R, Nishiyama H, Kobayashi T, Kawato M, Kanno T (2013). Downregulation of Ral GTPase-activating protein promotes tumor invasion and metastasis of bladder cancer. Oncogene.

[76] Minami N, Matsuura M, Yamamoto S, Honzawa Y, Yamada S, Koshikawa Y, et al. P012 Ral activation exacerbates colonic inflammation through the impairment of intestinal barrier function in experimental murine colitis. Eur Crohn’s Colitis Organ. 2017; [cited 2018 Nov 1];Poster abstract. Available from: https://www.ecco-ibd.eu/publications/congress-abstract-s/abstracts-2017/item/p012-ral-activation-exacerbates-colonic-inflammation-through-the-impairment-of-intestinal-barrier-function-in-experimental-murine-colitis.html.

[77] Diaz-Ochoa VE, Lam D, Lee CS, Chazin WJ, Skaar EP, Correspondence MR (2016). *Salmonella* mitigates oxidative stress and thrives in the inflamed gut by evading calprotectin-mediated manganese sequestration. Cell Host Microbe.

[78] Drumo R, Pesciaroli M, Ruggeri J, Tarantino M, Chirullo B, Pistoia C (2016). *Salmonella enterica* serovar Typhimurium exploits inflammation to modify swine intestinal microbiota. Front Cell Infect Microbiol.

[79] Chirullo B, Pesciaroli M, Drumo R, Ruggeri J, Razzuoli E, Pistoia C (2015). *Salmonella* Typhimurium exploits inflammation to its own advantage in piglets. Front Microbiol.

[80] Feng Z-Z, Jiang A-J, Mao A-W, Feng Y, Wang W, Li J (2018). The *Salmonella* effectors SseF and SseG inhibit Rab1A-mediated autophagy to facilitate intracellular bacterial survival and replication. J Biol Chem.

[81] Sarantis H, Grinstein S (2012). Subversion of phagocytosis for pathogen survival. Cell Host Microbe.

[82] Ibarra JA, Steele-Mortimer O (2009). *Salmonella*--the ultimate insider. *Salmonella* virulence factors that modulate intracellular survival. Cell Microbiol.

[83] Lindgren SW, Stojiljkovic I, Heffron F (1996). Macrophage killing is an essential virulence mechanism of *Salmonella* Typhimurium. Proc Natl Acad Sci U S A.

[84] Ainslie-Garcia MH, Farzan A, Newman JE, Friendship RM, Lillie BN (2018). Salmonella fecal shedding in pigs from birth to market and its association with the presence of Salmonella in palatine tonsils and submandibular lymph nodes at slaughter. Can J Vet Res.

[85] Reinhardt H, Shoveller AK, Farzan A, McBride B, de Lange CFM, Huber L (2019). Effect of nursery feeding program on serum haptoglobin, growth performance, and carcass characteristics of pigs reared on commercial farms. Can J Vet Res.

[86] Schut CH, Farzan A, Ainslie-Garcia MH, Friendship RM, Lillie BN (2019). Antibody responses to *Salmonella* in pigs from weaning up to marketing and presence of *Salmonella* at slaughter. Foodborne Pathog Dis.

[87] Chang CC, Chow CC, Tellier LC, Vattikuti S, Purcell SM, Lee JJ (2015). Second-generation PLINK: rising to the challenge of larger and richer datasets. Gigascience.

[88] Purcell S, Neale B, Todd-Brown K, Thomas L, Ferreira MAR, Bender D (2007). PLINK: a tool set for whole-genome association and population-based linkage analyses. Am J Hum Genet.

[89] Weir BS, Anderson AD, Hepler AB (2006). Genetic relatedness analysis: modern data and new challenges. Nat Rev Genet.

[90] Chen H, Wang C, Conomos MP, Stilp AM, Li Z, Sofer T (2016). Control for population structure and relatedness for binary traits in genetic association studies via logistic mixed models. Am J Hum Genet.

[91] Chen H, Conomos MP (2018). GMMAT: Generalized linear mixed model association tests. R package version 0.9.3.

[92] R Core Team (2018). R: A language and environment for statistical computing.

