# Supplementary material for: Identification of single-nucleotide variants associated with susceptibility to Salmonella in pigs using a genome-wide association approach
Source: BMC Vet Res. 2020 May 15;16:138. doi: 10.1186/s12917-020-02344-0 (PMC7227190; doi:10.1186/s12917-020-02344-0)
Supplement: Supplementary file 2 — Additional file 2: Table S1. Allele frequencies in case and control pig populations for SNVs with significant associations in GWAS models. [file 12917_2020_2344_MOESM2_ESM.docx]

Supplementary Table 1. Allele frequencies in case and control pig populations for SNVs with significant associations in GWAS models.

| Trait | SNV ID | | Gene^a^ | | GWAS  p-value | | Minor allele | | MAF^b^ in cases | | MAF in controls | | χ^2^  p-value | |
| --- | --- | --- | --- | --- | --- | --- | --- | --- | --- | --- | --- | --- | --- | --- |
| *Salmonella* seropositivity | rs81241392 | | *RALGAPA2* | | 7.60E-05 | | C | | 0.260 | | 0.148 | | 1.05 × 10^-6^ | |
|  | rs323410857 | | *RALGAPA2* | | 1.27E-04 | | T | | 0.256 | | 0.149 | | 1.36 × 10^-4^ | |
|  | rs81459294 | | *RALGAPA2* | | 2.09E-04 | | C | | 0.333 | | 0.214 | | 5.79 × 10^-7^ | |
|  | rs80868434 | | *RALGAPA2* | | 2.56E-04 | | A | | 0.354 | | 0.224 | | 1.26 × 10^-7^ | |
| *Salmonella* shedding | rs324041697 | | *PTPRJ* | | 6.60E-06 | | T | | 0.322 | | 0.524 | | 4.08 × 10^-14^ | |
|  | rs81476180 | | *ST6GALNAC3* | | 9.90E-06 | | A | | 0.306 | | 0.509 | | 2.85 × 10^-14^ | |
| *Salmonella* isolation at slaughter | rs322440805 | *DCDC2C* | | 1.60E-06 | | C | | 0.284 | | 0.516 | | 7.97 × 10^-10^ | |  |
|  | rs326411709 | *DCDC2C* | | 3.70E-06 | | A | | 0.291 | | 0.525 | | 7.37 × 10^-10^ | |  |
|  | rs319944764 | *DCDC2C* | | 8.60E-06 | | A | | 0.604 | | 0.391 | | 1.57 × 10^-8^ | |  |
|  | rs81348815 | | *AKAP12* | | 2.01E-05 | | A | | 0.279 | | 0.481 | | 8.11 × 10^-8^ | |
|  | rs80951933 | | *AKAP12* | | 3.05E-05 | | T | | 0.270 | | 0.474 | | 5.61 × 10^-8^ | |
|  | rs80903645 | | *AKAP12* | | 3.58E-05 | | T | | 0.252 | | 0.439 | | 4.72 × 10^-7^ | |
|  | rs80840697 | | *AKAP12* | | 4.10E-04 | | A | | 0.268 | | 0.430 | | 1.31 × 10^-5^ | |
| ^a^If the variant was intergenic, the closest gene within a 1 Mbp window was indicated.  ^b^MAF = minor allele frequency. | | | | | | | | | | | | | | |
